# Genetic algorithm as an optimization tool for the development of sponge cell culture media

**DOI:** 10.1007/s11626-018-00317-0

**Published:** 2019-02-11

**Authors:** Stephanie Munroe, Kenneth Sandoval, Dirk E. Martens, Detmer Sipkema, Shirley A. Pomponi

**Affiliations:** 1 Bioprocess Engineering, Wageningen University & Research, Wageningen, Netherlands; 2 Harbor Branch Oceanographic Institute, Florida Atlantic University, Boca Raton, FL USA; 3 Laboratory of Microbiology, Wageningen University & Research, Wageningen, Netherlands

**Keywords:** Genetic algorithm, Medium optimization, Sponge, Cell culture, *Dysidea etheria*, Metabolic activity

## Abstract

Sponges are rich sources of novel natural products. Production in cell cultures may be an option for supply of these compounds but there are currently no sponge cell lines. Because there is a lack of understanding about the precise conditions and nutritional requirements that are necessary to sustain sponge cells in vitro, there has yet to be a defined, sponge-specific nutrient medium. This study utilized a genetic algorithm approach to optimize the amino acid composition of a commercially available basal cell culture medium in order to increase the metabolic activity of cells of the marine sponge *Dysidea etheria*. Four generations of the algorithm were carried out in vitro in wet lab conditions and an optimal medium combination was selected for further evaluation. When compared to the basal medium control, there was a twofold increase in metabolic activity. The genetic algorithm approach can be used to optimize other components of culture media to efficiently optimize chosen parameters without the need for detailed knowledge on all possible interactions.

**Electronic supplementary material:**

The online version of this article (10.1007/s11626-018-00317-0) contains supplementary material, which is available to authorized users.

## Introduction

The discovery that marine sponges are a natural source for novel bioactive compounds has created an interest in the in vitro cultivation of their cells (Koopmans et al. 2009). Researchers have spent decades attempting to develop a cell line from multiple sponge species. These efforts have resulted in primary cell cultures with a finite lifespan (Pomponi and Willoughby 1994), but not in the development of a continuous cell line. One of the critical components in the development of any cell line is the nutrient medium in which the cells will be grown (Willoughby and Pomponi 2000). Various methods to optimize sponge nutrient media have been attempted. Willoughby and Pomponi (2000) used a one-dimensional approach to test the cellular response to different concentrations of fetal bovine serum, selenium, and glutamine. Other researchers have taken different approaches, using the Plackett-Burman design in combination with response surface methodology and the uniform design for multivariable medium optimization (Zhao et al. 2005). Different methods have been utilized to measure the cellular output, one of which is a colorimetric MTT assay to measure sponge cell responses to different nutrient medium components (Zhang et al. 2004). Regardless of the chosen method, there is still little known about the in vitro nutritional requirements for sponge cell proliferation and maintenance (Cai and Zhang 2014).

Nutrient media have a number of required components. An important group of components are the amino acids, which are fundamental for the synthesis of proteins and nucleotides. It has previously been observed that glutamine affected metabolic activity, esterase activity, DNA content, and protein content in cells of the sponges *Axinella corrugata*, *Hymeniacidon perleve*, and *Suberites domuncula* (Willoughby and Pomponi 2000; Zhang et al. 2004; Zhao et al. 2005). Analysis of the amino acid composition of the sponge *Amphimedon queenslandica* revealed that nine amino acids were at least twice as abundant in comparison to a typical mammalian hybridoma cell line (Watson et al. 2014). This suggests that nutrient media developed for mammalian cell culture may be suboptimal in the amino acids required by sponge cells.

An optimization tool known as the genetic algorithm (GA) has been utilized to optimize the growth of insect cells (Marteijn et al. 2002), microalgal cells (Camacho-Rodríguez et al. 2015), dinoflagellate cells (López-Rosales et al. 2015), and even for the optimization of the production of an aromatic compound by yeast (Etschmann et al. 2003). The GA offers an alternative method to the statistical factorial design, which is limited by the number of parameters being evaluated (Ranganath et al. 1999). It mimics the process of natural selection and allows for the simultaneous optimization of a large number of components with known or unknown effects on the cells, and inherently includes interaction effects that may impact the results (Marteijn et al. 2002).

The GA creates a population of “individuals” (in this study, medium compositions or “treatments”) and each “individual” is a unique combination of the different components that are selected to be part of the study. Each medium component is represented by a binary string (“gene”) and the different binary strings are combined on a “chromosome.” The value of the bit strings determines the component concentrations. In the present study, each “gene” codes for an amino acid concentration and the combination of the different “genes” forms a unique medium composition. Each “individual,” or unique medium composition, is evaluated for its fitness, which is metabolic activity in this study. The algorithm next selects the best performing “individuals” that will have the highest likelihood of being selected. Then, through random mutations on the selected individual “chromosomes” and recombination events between these “individuals,” a new population of “individuals” is created, of which at least some “individuals” are expected to perform better than the best “individuals” in the previous generation. A thorough description of the theory, principles, and mechanisms of the GA can be found in the literature (Ranganath et al. 1999; Weuster-Botz 2000; Marteijn et al. 2002).

This study utilized the GA to optimize the amino acid composition of a commercially available basal cell culture medium (M199) for the in vitro cultivation of cells of the marine sponge *Dysidea etheria*. This medium was used as a starting point since it has previously been shown to improve the metabolic activity of marine sponge cells (Pomponi et al. 1997). The data presented in this study represent a method to optimize the metabolic activity of cells of marine sponges or of other cell types where no commercially available medium is available.

## Materials and Methods

### Sample collection and preparation

Three individuals of *Dysidea etheria* were collected from the roots of mangroves (< 1.5 m depth) by snorkeling off of Summerland Key, FL, USA (24° 39′ 49.7” N, 81° 27′ 41.0” W) in July 2017. Salinity at the site, measured with a refractometer, was 34 ppm and the water temperature was 32.5 °C. Samples were processed immediately after collection, as described by Pomponi and Willoughby (1994). Sponges were cut into small pieces (~ 1cm^3^) with sterile scalpels in 0.2 μm filtered seawater (FSW). The minced sponge fragments were transferred to a sterile gauze pad and squeezed to release the cells. The cell suspension was filtered through a 40 μm cell strainer (Fisher, Hampton, NH) into a 50 ml centrifuge tube. Cells were concentrated by centrifugation at 500×*g* for 5 min. The supernatant was removed and cells were resuspended in FSW. Cells were counted with a disposable hemocytometer (INCYTO).

The dissociated cells were again concentrated by centrifugation in a microcentrifuge for 5 min at 500×*g*. The supernatant was removed and cells were resuspended in a cryoprotectant (10% dimethyl sulfoxide [DMSO] + 10% fetal bovine serum + 80% FSW) (Munroe et al. in press) to a final cell density of 1E+08 cells/ml. One to two milligrams of cells were transferred to 2.0 ml cryovials and placed in a Mr. Frosty (Thermo Scientific, Waltham, MA) to cool at 1°C/min in a – 80 °C freezer (Thermo Scientific) overnight before they were transferred into a cryobox for storage at – 80 °C.

Solutions of artificial seawater (ASW) and calcium-magnesium-free seawater (CMF) were prepared by dissolving salts into filter sterilized deionized water (DIW) and then autoclaving at 121 °C for 25 min. Solutions were then stored at 4 °C for further use. ASW was modified from Zhang et al. (2004) as follows: sodium chloride (23.3 g/L), magnesium chloride hexahydrate (10.2 g/L), sodium sulfate (1 g/L), calcium chloride (1.1 g/L), potassium chloride (1 g/L), trizma hydrochloride (4.02 g/L), and trizma base (2.97 g/L). CMF was prepared as follows: sodium chloride (28.55 g/L), sodium sulfate (1 g/L), potassium chloride (1 g/L), trizma hydrochloride (4.02 g/L), trizma base (2.97 g/L). All salts were purchased from Sigma Aldrich (St. Louis, MO).

For experiments, cells were removed from the – 80 °C freezer and the cryovials were immediately submerged in a water bath (50 °C) for 1–2 min until only a small ice crystal remained. Cells were then transferred to a microcentrifuge tube containing 500 μl of ASW and spun for 5 min at 4500 rpm (Eppendorf Mini Spin). The supernatant was removed and cells were resuspended in 1 ml of ASW and the process was repeated to rinse the cryoprotectant from the cell suspension. The final cell suspension was resuspended in 1 ml of CMF to prevent aggregation and was counted with a disposable hemocytometer.

### Marine Medium 199 (M199)

#### Media preparation

M199 (Sigma M3769) was used as a basal medium for all experiments. Marine M199 was prepared by adding ASW stock solutions to the M199 powder at the following concentrations: M199 (9.4 g/L), sodium chloride (17.25 g/L), magnesium chloride hexahydrate (10.04 g/L), sodium sulfate (0.88 g/L), calcium chloride (0.4 g/L), potassium chloride (0..25 g/L), trizma hydrochloride (4.02 g/L), trizma base (2.97 g/L), L-glutamine (0.025 g/L), rifampicin (0.03 g/L), and amphotericin B (0.003 g/L).

#### Stock solutions

Stock solutions of each of the 20 amino acids (AAs) were prepared by dissolving the AA into filter sterilized DIW and then filter sterilizing the solution through a 0.2 μm filter. All stocks were prepared at 5 g/L. The addition of 1 M sodium hydroxide was required to solubilize the aspartic acid and glutamic acid solutions, resulting in a final concentration of ~ 4.6 g/L. Stock solutions of the antibiotic rifampicin (100 g/L) and the antimycotic amphotericin B (10 g/L) were prepared by dissolving the powder in DMSO. All AAs and antimicrobials were purchased from Sigma Aldrich, except sodium hydroxide (Fisher). The stock solutions were subsequently aliquoted into smaller volumes and stored at – 20 °C for further use.

Stock solutions of the individual components of ASW were prepared by dissolving in filter sterilized DIW. The ASW stock solutions were autoclaved at 121 °C for 25 min. All stock solutions were aliquoted into appropriate volumes for storage at – 20 °C. Solutions were prepared at the following concentrations: sodium chloride (275 g/L), magnesium chloride hexahydrate (200 g/L), and sodium sulfate, calcium chloride, potassium chloride, trizma hydrochloride, and trizma base were prepared at 50 g/L.

#### Amino acid optimization via GALOP

GALOP (Version 2.20, Institute of Biotechnology in Jülich, Germany) was used to design and conduct four generations of GA experiments to optimize the AA composition. A maximum and minimum concentration for each AA was entered into the GALOP software as the upper and lower boundaries for each parameter with a step size of one. These boundaries were chosen from two preliminary experiments in which three generations of the GA were used to optimize 10 AAs simultaneously. The lowest and highest concentrations of each AA from the top five treatments from both preliminary experiments were selected (Table 1). The concentrations were then converted into a corresponding volume of stock solution to be added. Metabolic activity was selected as the target function to optimize and was given a relative weight of one. GALOP software gives the option to select optimal bit length and this function was utilized for all 20 AAs, resulting in bit lengths between three and six (Table 1). Bit length is determined by the number of steps between the maximum and minimum value (ex: a bit length of four results in 2^4^ = 16 values within the concentration range). In each generation, the population size was fixed at 30 unique medium compositions (referred to as “treatments”) to maximize the capacity of a 96-well plate, and each treatment was performed in triplicate. The initial generation was randomly generated and each subsequent generation was executed utilizing the roulette wheel option, and a 95% probability for crossing over and a 2% mutation rate were chosen. Normalized relative fluorescent units (RFUs) of the metabolic activity were obtained using the microplate reader and were entered as results into the GA, which were then used to determine the next generation.

**Table 1:** Initial concentration range used in generation one for the amino acids optimized in the GA. The concentration of each AA in the basal Marine M199 is also shown and each upper and lower concentration of the AA includes the basal concentration

Parameter	Marine M199 concentration (μg/ml)	Lower concentration (μg/ml)	Upper concentration (μg/ml)	# steps
l-Alanine	25	25	140.6	37
l-Arginine·HCl	70	73.13	138.75	21
l-Aspartic Acid	30	41.5	127.75	30
l-Asparagine	0	78.13	121.88	14
l-Cysteine·HCl·H_2_O	0.11	25.11	121.99	31
l-Cystine·2HCl^a^	26	26	26	0
l-Glutamic Acid	66.8	78.3	130.05	18
l-Glutamine	25	84.38	100	5
Glycine	50	50	90.63	13
l-Histidine·HCl·H_2_O	21.88	121.88	143.76	7
Hydroxy-l-Proline^a^	10	10	10	0
l-Isoleucine	20	25	91.88	15
l-Leucine	60	63.13	147.5	27
l-Lysine·HCl	70	82.5	120	12
l-Methionine	15	43.13	127.5	27
l-Phenylalanine	25	96.88	134.38	12
l-Proline	40	43.13	136.88	30
l-Serine	25	78.13	143.75	21
l-Threonine	30	33.13	117.5	27
l-Tryptophan	10	10	88.13	25
l-Tyrosine·2Na·2 H_2_O	57.66	73.29	104.54	10
l-Valine	25	62.5	143.8	26

^a^Component present in Marine M199, but was not added in the GA experiments

Each treatment was prepared by reducing the volume of DIW added to Marine M199 so that by adding the volume of each AA designated in the GA, a final solution with the correct concentration of each component was obtained. Example tables are available in the supplemental materials. Each treatment was divided into two portions, one containing *D. etheria* cells at a final concentration of 6E+06 cells/ml and a control which contained an equal volume of CMF. Two hundred microliters of medium + cells or medium controls were incubated in two black 96-well plates with clear bottoms (Corning, Corning, NY). Three replicates of each of the 30 medium treatments were cultured for 48 h in the dark at 20 °C.

The intracellular metabolic activity of the cultures was assessed with the Vybrant Cell Metabolic Assay Kit (ThermoFisher V23110) according to the manufacturer’s instructions. Cells were centrifuged in the microplate, the supernatant was removed, and the cells were resuspended in an equal volume (200 μl) of a 10 μM working solution of C_12_-resazurin prepared in ASW. The cells were then incubated at room temperature for 15 min in the dark. The microplates were centrifuged for 5 min at 350×*g* (Thermo Scientific Sorvall Legend X1R), the supernatant removed, and the cells resuspended in 200 μl of ASW. Fluorescence was measured with a BMG LABTECH NOVOstar microplate reader (ex/em 570/620-10 nm, gain 3106). In order to normalize the data so that comparisons could be made between generations, a relative metabolic increase value was calculated by dividing the C_12_-resazurin RFU (and its associated standard deviation) for each treatment by the Marine M199 (control) RFU. A value for the relative increase in metabolic activity (RIMA) greater than one indicates that an improvement over the control was obtained from that particular treatment. Cells were also imaged with a fluorescent microscope (EVOS Auto FL, ThermoFisher Scientific), using a Texas Red filter, to verify plate reader results.

#### Optimized treatment comparisons

The top two treatments from generation 4 were evaluated more extensively with primary cell cultures from three individuals of *D. etheria* in wet lab conditions to determine which treatment resulted in the highest metabolic activity*.* Medium was prepared as previously described. Triplicate cultures for both of the treatments and their respective cell-free controls, as well as Marine M199 and ASW controls, were incubated in 96-well plates with clear bottoms for each of the three sponge individuals for 48 h in the dark at 20 °C. Cultures were then stained with the metabolic activity stain, as previously described, and the microplates were read on a NOVOstar plate reader (ex/em 570/620-10 nm, gain 3106). Cells were imaged with an EVOS fluorescent microscope using the Texas Red filter.

Viability of the three sponge individuals was assessed using the fluorescent stain 7-AAD (ThermoFisher Scientific A1310) after the cells were thawed. Cells were stained according to manufacturer’s instructions and microscopically counted using a disposable hemocytometer. Images were taken with an EVOS fluorescent microscope using the Cy5 filter and the percentage of dead cells was assessed.

#### Statistical analyses

The average RFU and standard deviation for metabolic activity measurements were calculated for all treatments. A normalized value was calculated for all treatments by subtracting fluorescent RFU readings of the controls from the treatments.

A student’s *t* test was used to evaluate significant differences in metabolic activity between the Marine M199 control and all treatments in each generation of the genetic algorithm as well as for the *optimized treatment comparisons* experiment. A one-way ANOVA was performed in Excel to determine if there was significant variation between sponge individuals for the *optimized treatment comparisons* experiment. Each individual of *D. etheria* was evaluated separately in the *optimized treatment comparisons* experiment.

## Results

### Amino acid optimization via GALOP

**Fig. 1:**
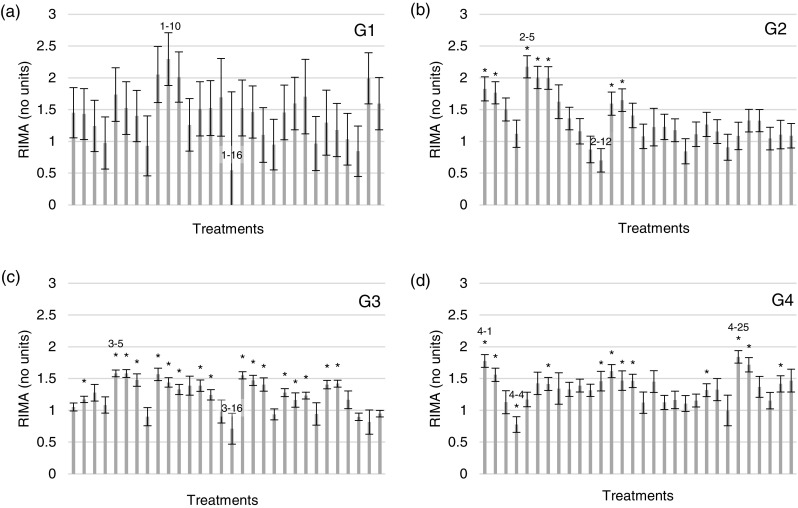
Relative increase in metabolic activity (RIMA) for each treatment of G1 (***a***), G2 (***b***), G3 (***c***), and G4 (***d***). Data are displayed as the normalized average of three replicates of each treatment divided by the Marine M199 control ± SD. * indicates a significant improvement (*p* < 0.05) over Marine M199. Treatments labeled 1-10, 1-16, etc. represent the highest and lowest metabolic activities for each generation and correspond with Fig. 2.

In this study, the objective was to optimize the concentrations of AAs in order to maximize the metabolic activity of cells from *D. etheria* in wet lab conditions. The first generation (G1) resulted in 27 of 30 treatments with improved cellular metabolic activity when compared to the Marine M199 control; however, none of these treatments was significantly improved (Fig. 1*a*). In the second generation (G2), 26 of 30 treatments had improved cellular metabolic activity, seven of which were significantly improved (*p* ≤ 0.05) when compared to the control (Fig. 1*b*). The third generation (G3) resulted in 22 of 30 treatments with improved cellular metabolic activity and 16 of these treatments were significantly improved when compared to the control (Fig. 1*c*). In the fourth and final generation (G4), 28 of 30 treatments were improved, 11 of which were significantly improved when compared to the control (Fig. 1*d*).

When G2, G3, and G4 were compared to G1, the relative increase in metabolic activity (RIMA) did not increase; however, the variation between replicates consistently decreased, resulting in more treatments that were significantly improved. In addition, the AA concentration ranges were narrowing in G3 and G4, indicating that convergence upon an optimal concentration was occurring. Results clearly indicate that the intracellular metabolic activity of *D. etheria* cells increases when Marine M199 was supplemented with additional AAs. The genetic algorithm was terminated after four generations because only incremental improvements were observed and metabolic activity was similar between treatments. All cultures from each generation were monitored microscopically and no contaminants were detected.

**Fig. 2:**
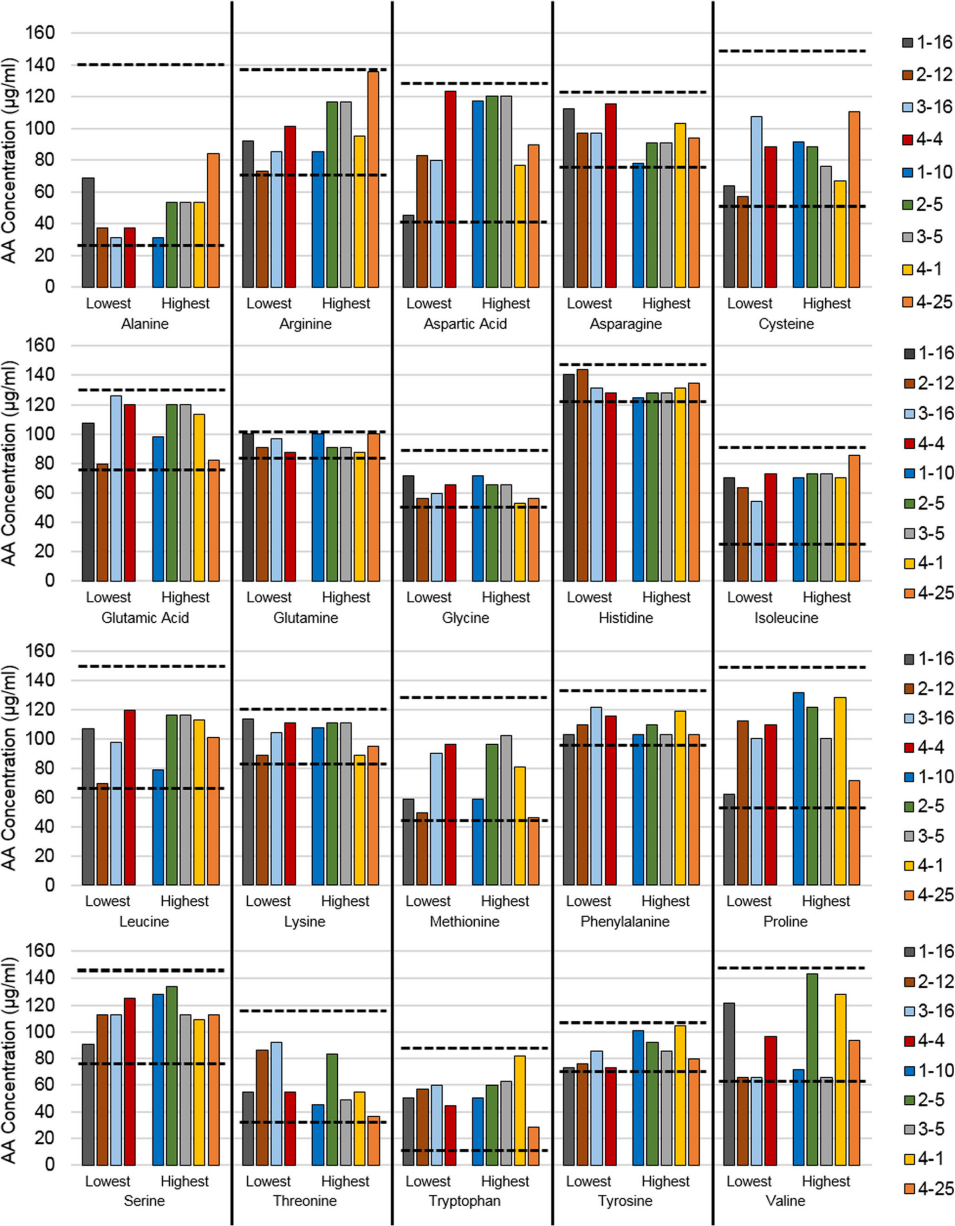
Comparison of AA concentrations for the treatments with the highest and lowest relative increase in metabolic activity from each generation. AAs are alphabetical in graphs ***a*** alanine through cysteine, ***b*** glutamic acid through isoleucine, ***c*** leucine through proline, and ***d*** serine through valine. Vertical solid bars separate each AA and horizontal dashed bars represent the minimum and maximum concentration allowed for each AA in the GA. The legend shows the generation followed by the treatment number (ex: 1-10 is generation one—treatment 10).

The amino acid compositions of treatments with the highest and lowest metabolic activities in each generation were compared and the minimum and maximum concentration for each AA are displayed to determine whether the AA was consistently approaching the minimum or maximum values within the chosen concentration range. (Fig. 2). Results indicate that there was not a strong correlation between individual AAs and their corresponding metabolic activities; however, there was a weak positive correlation in response to arginine. No treatments displayed a high AA concentration with a high metabolic activity and a low AA concentration with a low metabolic activity (or vice versa). The top two treatments (when compared to the control) from G4 (treatment 4-1 *p* = 0.0044; treatment 4-25 *p* = 0.0039) were selected for further evaluation.

### Optimized treatment comparisons

**Fig. 3:**
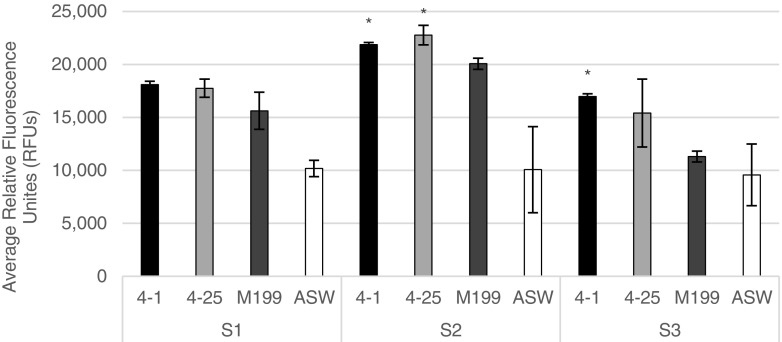
Metabolic activity of three *D. etheria* individuals (S1, S2, and S3) incubated in 4-1 and 4-25 from G4, Marine M199, and ASW controls. Data are displayed as the average of three replicates ± SD; *significant improvement (*p* < 0.05) over M199.

Cells from three individuals of *Dysidea etheria* were tested in these two treatments to verify that the responses to the treatments were not specific to one sponge individual. There was significant variation in metabolic activity between the three sponge individuals for treatment 4-1 (*p* = 0.0345), treatment 4-25 (*p* = 0.0254), and Marine M199 (*p* = 0.0003) (Fig. 3). Therefore, data were analyzed separately for each sponge individual. There was no significant variation between individuals for the ASW control (*p* = 0.9757).

Although there was variation between sponge individuals, the same trend is seen for 4-1, 4-25, and Marine M199 for each sponge individual (Fig. 3). Sponge two (S2) consistently has the highest metabolic activity while sponge three (S3) consistently has the lowest. This was in agreement with the viability measurements from cryopreserved cells that showed that S2 had the highest viability (96.39%), followed by sponge one (S1, 95.84%), and S3 (88.72%). All sponges show the same trend with the optimized treatments being better than M199, and M199 being better than ASW. There were significant differences between 4 and 1 and Marine M199 (Fig. 3) for both S2 and S3, while 4-25 was only significantly improved over Marine M199 in S2. In S1, neither 4-1 nor 4-25 were significantly improved.

## Discussion

The strength of the genetic algorithm for medium optimization lies in its ability to optimize many media components simultaneously while accounting for the interaction effects that the components may have on one another (Camacho-Rodríguez et al. 2015). It is for this reason that the GA is particularly well suited for developing a culture medium with many components for cells for which little is known about their metabolism or nutrient requirements. This study utilized a GA approach to optimize the AA composition of Marine M199 in order to improve the in vitro metabolic activity of primary cell cultures of the marine sponge *Dysidea etheria.* Previous attempts to optimize specific nutrients, such as inorganic salts, amino acids, sugars, vitamin C, selenium, and animal serum, while utilizing a factorial design have been made (Willoughby and Pomponi 2000; Zhao et al. 2005), resulting in improvements to cell number, viability, or metabolic activity.

It is well documented that different sponge species respond in varying ways to basal media, but also to other components in the media (Pozzolini et al. 2014). Zhang et al. (2004) optimized glutamate, pyruvate, sodium citrate, and silicon for the sponge *Suberites domuncula* and when the same concentrations of nutrients were used with the sponge *Hymeniacidon perleve*, the results differed and it was determined that the effects of each nutrient are likely species dependent (Zhao et al. 2005). Because of the strong species-dependency, AA concentration ranges were evaluated independently of previous primary sponge cell culture studies where glutamine, aspartic acid, and glycine concentrations were assessed (Willoughby and Pomponi 2000; Zhao et al. 2005); however, previous concentrations were taken into consideration for the preliminary GA studies (data not shown). The requirements for the other AAs have not been previously studied in nutrient media for sponges.

The AA composition of the treatments with the highest and lowest metabolic activity from each generation of the GA were compared in further wet lab experiments to determine if the algorithm resulted in convergence towards an optimal concentration for each AA (Fig. 2). If all components are important, each of the AAs would converge on an ideal concentration. However, if a component is not as vital, that particular AA concentration might not make a significant contribution to the overall metabolic activity, and the concentration might vary among treatments. The individual AA concentrations do not need to be similar to one another, but the treatments which result in the highest increases in metabolic activity would be expected to have an overall composition similar to one another. Our results demonstrate that the ratio of AA concentrations within each of the treatments did not correlate with either the highest or lowest metabolic activities (Fig. 2). Although *D. etheria* cells did have significant increases in their metabolic activity, only arginine and tyrosine emerged as having a weak positive correlation to this increase.

Previous studies have documented that glycine (Shinagawa et al. 1992) and alanine are the most abundant AAs in the skeletal composition (Watson et al. 2014) of multiple sponge species and that the dry weight cellular fraction is higher in isoleucine, leucine, and valine in *Amphimedon queenslandica* (Watson et al. 2014). The skeletal component of sponges is made up of a collagen-based extracellular matrix (Watson et al. 2014). The AAs which are keys to the collagen triple-helix shape are glycine, alanine, and proline (Nelson et al. 2008) and should therefore be intrinsically present in sponges at higher amounts at both the skeletal and cellular level.

**Fig. 4:**
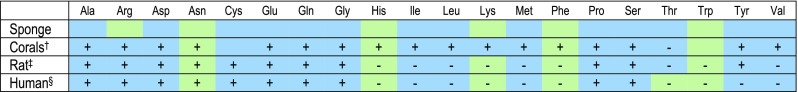
Survey of the proposed AA biosynthetic capabilities of two marine invertebrate and two vertebrate species, based on KEGG-pathways (Kanehisa and Goto 2000; Kanehisa et al. 2016, 2017). Blue squares indicate that the amino acid synthesis pathway is complete; green squares indicate that the pathway is not complete. Additional data from radiolabeled amino acid precursor studies and feeding studies (referenced in the footnote) are indicated by a “+” (AA can be synthesized) or “−“ (AA cannot be synthesized). If no data are available, the square is blank

While Andrade et al. (1999) used radiolabeled amino acid precursors to determine the biosynthetic origins of a natural compound produced by the sponge, comprehensive studies on the biosynthesis of AAs by sponges have not been conducted. Therefore, comparisons of the AA synthetic capabilities between two marine invertebrates (the sponge *Amphimedon queenslandica* and five species of scleractinian corals) and two vertebrate species were made using the KEGG PATHWAY database for biosynthesis of AAs (Fig. 4), which consists of reference pathway maps for each of the 20 AAs with organism-specific pathways generated from reference genomes (Kanehisa et al. 2017). Additionally, feeding studies and radiolabeled AA precursor studies were incorporated (see references in Fig. 4) to determine if the proposed pathways in KEGG align with the actual synthetic capabilities of the organisms.

Based on the KEGG Pathways, the sponges that were evaluated in these studies are not capable of synthesizing arginine, asparagine, histidine, lysine, phenylalanine, and tryptophan (Fig. 4). The AA optimized medium that resulted from four generations of the GA (4-1) had the highest concentrations of histidine, valine, proline, and phenylalanine and the lowest concentrations of alanine, glycine, and threonine (Fig. 2). The threonine pathway is not complete for all organisms included in the KEGG Pathway studies, and both rats and humans were incapable of synthesizing threonine during feeding studies (Fig. 4). At least one species of sponge (*Amphimedon queenslandica*) lacks the pathways for synthesizing histidine and phenylalanine (based on KEGG Pathways) and they are essential AAs for rats and humans; however, coral fragments were capable of biosynthesizing both histidine and phenylalanine despite lacking the complete pathways in KEGG (Fig. 4). Just because the AA pathway is complete (or incomplete) in KEGG does not definitively answer whether or not the organism will be capable of its biosynthesis in vivo, as evidenced by complete KEGG pathways for some of the essential AAs that cannot be synthesized by humans, such as isoleucine, leucine, methionine, tyrosine, and valine (Fig. 4, Kanehisa et al. 2017). While proline and valine were supplied at the highest concentrations in the AA optimized medium (Fig. 2), the pathways for synthesis are present in *A. queenslandica*. This could indicate that there might be a limited capacity for synthesis during cell culture (Eagle 1959). It is also possible that proline is required in a higher amount because it is one of the major components of the collagen triple-helix (Nelson et al. 2008) and alanine and glycine are not limited by their biosynthetic capacities while proline must be supplemented from external sources. Additionally, valine is an essential amino acid in all vertebrates, but the coral fragments were capable of synthesizing valine (Fig. 4).

Sponges (represented by a single species, *A. queenslandica*) were the only “group” in Fig. 4 lacking the synthetic capability to produce arginine; however, in the current study, there was a weak positive correlation between the addition of arginine and increases in metabolic activity. Further analysis of nine other marine invertebrates and three other vertebrates in the KEGG-Pathway showed that they were all capable of arginine biosynthesis. Results not included in Fig. 4 found that nematodes, molluscs, crustaceans, insects, and fishes cannot synthesize arginine even though the pathway is present (Fitzgerald and Szmant 1997). This could indicate that sponges also require arginine as an essential AA. Although treatments with higher arginine concentrations tended to result in a higher metabolic activity, this was not always true since some of the treatments with the lowest metabolic activities also contained high arginine concentrations (Fig. 2). Further studies will ultimately be necessary to determine whether each of the AAs is essential or non-essential in sponges; however, this study has provided some preliminary insight into which of the AAs might fall into each category.

During this study as well as previous studies (Willoughby and Pomponi 2000; Munroe et al. in press), it has been documented that individual variation occurs within many species of sponges. The objective of the *optimized treatment comparisons* test was to verify the results of the GA experiments by further evaluating two additional sponge individuals, which confirmed the high individual variation within *Dysidea etheria*. When the three individuals were cultured in ASW the results were comparable, but they responded differently to medium treatments with S2 having the highest metabolic response. It is therefore possible that the differences in response to the AA medium could be due to factors such as reproductive or filtration states or stochastic variation between individuals; however, it remains that the three individuals all demonstrated increases in metabolic activity in response the addition of amino acids to the medium (4-1 and 4-25 > M199 > ASW). Many other nutritional components commonly found in nutrient media, such as lipids, vitamins, and trace elements, are likely important components for sponge cell culture (De Caralt et al. 2007), but were not evaluated in the present study, and further optimization of additional components such as vitamins, trace elements, growth factors, and serum are in progress.

## Conclusions

The use of the genetic algorithm for the optimization of AAs in nutrient media resulted in the creation of media compositions with differing AA concentrations. Some of these unique media compositions resulted in significant improvements to the metabolic activity of primary cell cultures of *D. etheria*. Trends of increasing metabolic activity in response to increasing arginine concentrations as well as the absence of a complete arginine biosynthetic pathway in the KEGG model indicate that arginine is likely an essential amino acid for sponges; however, there were not strong correlations between the remaining 19 amino acids and the metabolic activity in either a positive or negative manner. This study has taken a necessary first step towards optimizing the nutritional requirements for primary cell cultures of the marine sponge *Dysidea etheria* in wet lab conditions, but did not yet result in clear increases in cell number*.* As more knowledge becomes available about biological factors, GA studies can be designed to optimize the culture conditions for each of these parameters. This approach provides a simplified method that is suitable for optimization of a relatively large number of medium components for non-model organisms for which nutrient requirements and potential interactions of nutrients are unknown. The GA approach to medium optimization can be utilized for other marine invertebrate cell cultures, and may be useful in the future to increase production of bioactive compounds of interest from a sponge cell line.

## Electronic supplementary material

ESM 1 (PDF 181 kb)
