# Supplementary material for: Genetic algorithm as an optimization tool for the development of sponge cell culture media
Source: In Vitro Cell Dev Biol Anim. 2019 Feb 11;55(3):149–58. doi: 10.1007/s11626-018-00317-0 (PMC6407725; doi:10.1007/s11626-018-00317-0)
Supplement: Supplementary file 1 — (PDF 181 kb) [file 11626_2018_317_MOESM1_ESM.pdf]

Supplementary Materials

Table 2 The total volume of amino acids added to each treatment. This varies between generations as the algorithm makes improvements and changes to each subsequent generation and is an example from G1. The volume of Marine M199 always stays the same and the addition of DIW keeps the concentration of all components in Marine M199 the same in every treatment.

Treatment	Total AA Vol	M199 Vol	DIW Vol	Total Vol
1	348	1055	97	1500
2	311	1055	134	1500
3	385	1055	60	1500
4	336	1055	109	1500
5	414	1055	31	1500
6	401	1055	44	1500
7	379	1055	66	1500
8	360	1055	85	1500
9	356	1055	89	1500
10	338	1055	107	1500
11	391	1055	54	1500
12	395	1055	50	1500
13	326	1055	119	1500
14	354	1055	91	1500
15	406	1055	39	1500
16	326	1055	119	1500
17	366	1055	79	1500
18	337	1055	108	1500
19	426	1055	19	1500
20	368	1055	77	1500
21	333	1055	112	1500
22	377	1055	68	1500
23	362	1055	83	1500
24	363	1055	82	1500
25	364	1055	81	1500
26	396	1055	49	1500
27	397	1055	48	1500
28	387	1055	58	1500
29	356	1055	89	1500
30	363	1055	82	1500
Control	0	1055	445	1500

Table 1 GALOP G1 output table for each AA. The three letter abbreviation is used for each of the 20 AA. Each value is the volume of each AA to be added to that treatment.

Treat-ment	Ala	Arg	Asp	Asn	Cys	Glu	Gln	Gly	His	Ile	Leu	Lys	Met	Phe	Pro	Ser	Thr	Trp	Tyr	Val
1	0	8	15	33	17	14	20	1	35	16	24	6	21	30	25	27	17	7	9	23
2	0	2	15	28	15	5	21	8	38	12	7	11	19	34	6	27	16	19	12	16
3	17	12	17	39	30	15	21	9	34	22	5	8	14	35	8	36	19	2	12	30
4	4	1	17	31	14	4	21	2	39	14	3	6	12	27	23	35	17	17	11	38
5	32	9	32	31	28	14	21	3	33	16	27	14	21	30	20	29	26	4	12	12
6	11	17	22	30	37	16	19	1	39	15	15	10	17	29	27	32	14	6	14	30
7	9	15	29	29	20	17	21	5	34	17	18	13	25	27	20	28	18	15	6	13
8	11	14	14	32	14	16	23	1	38	17	9	8	24	25	9	30	23	22	7	23
9	26	5	6	31	13	22	20	9	39	19	2	13	24	25	9	28	27	7	8	23
10	2	5	28	25	25	10	24	7	33	16	6	12	14	25	26	33	5	13	14	15
11	27	9	14	36	19	10	22	2	33	23	25	6	19	29	11	22	15	22	12	35
12	19	21	19	37	32	6	21	11	34	17	8	13	15	31	11	36	19	22	7	16
13	11	9	6	30	11	18	23	6	34	12	5	12	16	34	9	18	7	22	11	32
14	10	5	24	25	26	6	21	7	34	17	15	13	24	34	13	36	2	0	10	32
15	4	11	30	37	33	20	22	9	37	9	7	9	22	26	20	30	8	24	15	33
16	14	7	5	36	16	13	24	7	38	16	15	14	14	25	4	21	8	13	5	31
17	24	9	6	33	22	9	22	5	33	19	9	15	25	30	10	29	22	12	10	22
18	19	2	25	31	13	20	19	11	32	9	17	10	11	25	8	36	3	12	6	28
19	26	16	33	37	36	17	23	8	38	17	21	14	23	26	14	26	5	6	6	34
20	9	18	9	37	13	19	23	8	37	18	7	4	10	35	21	36	22	5	11	26
21	32	11	9	38	15	13	20	2	36	13	13	8	10	25	16	28	6	16	9	13
22	22	17	10	30	12	12	20	7	34	18	21	5	18	26	9	32	25	19	6	34
23	10	2	17	25	27	16	21	4	33	20	24	8	15	34	15	36	9	21	8	17
24	22	16	16	31	30	19	20	3	35	15	12	11	24	32	7	28	2	11	7	22
25	33	5	20	31	21	17	19	0	36	12	4	9	10	31	9	23	23	23	12	26
26	25	21	23	31	15	16	23	4	38	9	12	14	26	23	26	29	8	11	11	31
27	25	9	8	27	39	16	22	5	35	19	23	7	20	31	27	19	12	8	14	31
28	33	9	7	26	34	18	23	9	32	15	11	11	21	25	14	30	19	21	9	20
29	18	9	5	32	21	9	20	3	36	19	20	13	24	26	16	37	9	11	5	23
30	4	21	10	25	27	13	24	3	35	21	21	11	11	31	27	19	19	13	9	19
Control	0	0	0	0	0	0	0	0	0	0	0	0	0	0	0	0	0	0	0	0
